# A New Perspective of COVID-19 Infection: An Epigenetics Point of View

**DOI:** 10.1055/s-0041-1736565

**Published:** 2021-10-19

**Authors:** Ziya Ozturkler, Rasime Kalkan

**Affiliations:** 1 Ministry of Education and Culture, Higher Education and Foreing Affairs Department, Turkish Republic of Northern Cyprus, Nicosia, Cyprus; 2 Department of Molecular Biology and Genetics, Faculty of Arts and Sciences, Near East University, Nicosia, Cyprus; 3 Department of Medical Genetics, Faculty of Medicine, Near East University, Nicosia, Cyprus

**Keywords:** COVID-19, epigenetics, methylation

## Abstract

Coronavirus disease 2019 (COVID-2019) started in Wuhan, China, in December 2019. Angiotensin-converting enzyme 2 (ACE2) receptor was one of the most important genes related to the entrance of the virus to the host. Until now, several variations have been identified in ACE2 and related transmembrane protease serine 2. Epigenetic modifications not only play an important role during the maintenance of genome and cellular homoeostasis but also for the etiopathophysiology of the virus infection. Studies showed methylation of ACE2 was changed to depend on host and age of the host during the viral infection.

In this study, we provided an epigenetics point of view to the coronavirus infection. We highlight the importance of epigenetic modifications during viral replication and infection and their interaction with COVID-19 susceptibility and host viral response.

## Key Points

- Genetic and epigenetic profile play a key role during coronavirus disease 2019 (COVID-19) infection.
- Epigenetic alterations on the host genome are related to COVID-19 susceptibility.
- Genetic and epigenetic alterations in *ACE2, TMPRSS2, IFN* -related genes, and *FURIN* genes were an important determinant of severe acute respiratory syndrome coronavirus 2 cell entry of host and severity of the disease.

## Introduction

Coronaviruses (CoV) are a major family of pathogens that include severe acute respiratory syndrome coronavirus (SARS-CoV) and Middle East respiratory syndrome coronavirus (MERS-CoV) strain. 1 SARS-CoV-2 is a member of β-genus coronaviruses containing a single-strand enveloped RNA that is 29 Kb long. The similarity between SARS-CoV-2 and MERS-CoV was 50 and 80% with SARS-CoV. 2 SARS-CoV-2 infection caused coronavirus disease 2019 (COVID-19) in humans. The rapid spread of SARS-CoV-2 highlights the importance of understanding its nature and biological function. Studies showed that genetic and epigenetic modifications were important during the regulation of host response. 2 Epigenetic modifications are posttranslational chemical changes that occur at the level of histone proteins, RNA and DNA, and regulate gene expression without alterations of nucleotide sequence. 2 According to the RNA sequencing studies, 41 RNA modifications sites on viral transcripts were observed throughout the viral genome. These internal modifications contribute to viral RNA stability and translation efficiency and can be used for the explanation of escaping from host immune response. 2

In this article, we discussed genetic and epigenetic alterations that play a role during the SARS CoV-2 infection and show differences in tissue/cell-, age-, and sex-biased patterns. 2

## Genetic Alterations and SARS-CoV-2 Infection

The virus uses the angiotensin-converting enzyme 2 (ACE2) type I membrane receptor to enter the host cell. ACE2 receptors were found on different tissues like arterial and venous endothelial cells, alveolar cells, enterocytes of the small intestine, and arterial smooth muscle cells in most organs. 2 While the virus enters the host cell, subgenomic RNAs were transcribed from the genomic RNA that encodes the spike protein (S), an envelope protein (E), membrane protein (M), and nucleocapsid protein (N). 2 This encoded protein was a functional receptor for the human coronaviruses SARS, human coronavirus NL63 , and SARS-CoV-2. 3 Transmembrane serine protease 2 (TMPRSS2), cathepsin L (CTSL), and furin, paired basic amino acid cleaving enzyme (FURIN) were responsible for cleaving of SARS-CoV proteins. 2 The human *ACE2* gene contains hot spots for virus binding and mutations occur near these hot spots that were important to the host diversity of the virus. 2 4 5 Studies showed that N501 T mutation at the position significantly enhances the binding capacity of SARSCoV-2 spike protein receptor-binding domain. 6 Up to date, more than 1,700 variants have been identified on the *ACE2* gene, which is nonsense, missense, and intron variants in the 3–UTR of the gene. The important point of *ACE2* variants may affect intermolecular interaction with the SARS-CoV-2 S protein like interaction-booster between ACE2 and S1 or interaction-inhibitor between ACE2 and S1. 3

The *TMPRSS2* gene encoded a serine protease family member and is critical for the entry of viruses. The TMPRSS2 gene encodes a serine protease family member and is critical for the entry of viruses. Because this protease proteolytically cleaved and activated viral envelope glycoproteins during the infection. *TMPRSS2* polymorphisms were associated with an increased risk of severe COVID-19. 3 There is no published epigenetic study that shows the interaction *TMPRSS* gene and COVID-19. Methylation studies of the TMPRSS gene were based on prostate cancer patients. 3

The *FURIN* gene was located on chromosome 15q26.1 and plays an important role during the process of protein trafficking of the secretory pathway. Papa et al demonstrated that SARS-CoV-2 replication promoted by cleavage of furin. 7 *ADAM-17* gene plays a role during viral infection. The virus uses the angiotensin-converting enzyme 2 (ACE2) type I membrane receptor to enter the host cell. 7

An increased number of studies demonstrated that variations in *TMPRSS2, FURIN,* and *ADAM-17* may also have a role in the SARS-CoV-2 infectivity, disease severity, the outcome of the disease, and during the personalization of treatment planning of affected patients. Overall, an increased number of molecular profile studies will help us to better understand the heterogeneity of infection and disease and help us design personalized medicine tools on COVID-19 infection.

## Interaction between Epigenetic Modifications and *ACE2*

The *ACE2* gene is located on chromosome Xp22.2. The X-chromosome is one of the important chromosomes in the female that is epigenetically regulated. X-chromosome inactivation is an epigenetics process and regulated by X inactivation center). Because of random X inactivation, females are mosaic but males are hemizygous. 2 8 X inactivation is not complete and nearly 25% of X-chromosomal genes are escaped from X inactivation. *ACE2* was one of the genes that escape from X inactivation. 9 Epigenetic regulation of the *ACE2* gene was one of the mechanisms related to COVID-19 infection. Studies showed gender and age-dependent DNA methylation of the *ACE2* gene in airway epithelial cells. 10 DNA methylation level was decreased during aging and led to differential methylation patterns of several genes including aging and immune response-related genes. Therefore, DNA methylation is one of the main mechanisms that affects the prognosis of patients affected by SARS-CoV-2. 2 Under the cell energy stress, NAD-dependent histone deacetylase Sirtuin 1 (SIRT1) regulates ACE2. Also, upregulation was identified in the lung of patients with severe COVID-19. 4 In this point of view, we can suggest that increased expression level of ACE2 was correlated with severe COVID-19 infection.

Corley et al analyzed genome-wide methylation profiles of nine terminally ill COVID-19 patients' peripheral blood. They determined differentiated DNA methylation signature of severe COVID-19 that showed hypermethylation of IFN-related genes and hypomethylation of inflammatory genes that includes a regulatory region of the *NLRP3* inflammasome and antiviral *MX1* genes. The methylation pattern of *MX1* was associated with plasma SARS-CoV-2 viral load and platelet count. 11 Inactivation of the *IFN* gene and increased level of chemokine/cytokine gene expression patterns was observed during the SARS-CoV-2 infection. Therefore, studies concluded that SARS-CoV-2 suppressed the innate antiviral response of the host. 1 Alterations of IFN-stimulated genes, antigen presentation genes, and proinflammatory genes were identified on RNA-Seq studies from patients infected with SARS-CoV-2. 12 13

Corley et al demonstrated epigenetic alterations were changed depending on the cell type. According to the study, enhancer regions of primary neutrophils from peripheral blood were hypomethylated, but on the other side transcription start site regions of primary T cells, primary T helper cells, and primary T regulatory were hypermethylated. 11 Based on Corley's study, aberrant DNA methylation pattern at cell-type-specific regulatory regions of the host genome was observed on severe COVID-19.

RNA type viruses, like SARS-CoV-2, may also be sensitive to RNA modifications, including N6-methyladenosine (m6A) and N6,2′-Odimethyladenosine (m6Am) modifications (m6A/m). Identification of the importance of these RNA modifications will help us to explain the viral life cycle, host response, and severity of COVID-19. These studies showed the importance of further studies in the field of epigenetics will help us to understand the severity of the infection and host defense of SARS-CoV-2. A full understanding of the epigenetic background of COVID-19 will promote docking studies to discover possible targets on SARS-CoV-2 as an epigenetic-targeted agent of COVID-19. This will bring up the combination therapy of epigenetic and antiviral drugs for viral infection.

## Conclusion

Epigenetic changes regulate many normal and disease-related processes including cancer, imprinting disorders, obesity, and viral infections. Epigenetic modifications can be occurred posttranslationally in different ways that alter interactions with DNA and nuclear proteins and lead to changes in chromatin architecture and alterations in gene expression.

In general, hypomethylation of inflammatory genes, hypermethylation of IFN-related genes, and perturbations to the epigenetic clock and epigenetic inferred mortality risk were the major epigenetic alterations of severe COVID-19 cases. Studies showed that SARS-CoV-2 alters host epigenome in various ways. Genetic and epigenetic alterations in *ACE2, TMPRSS2,* IFN-related genes, and *FURIN* genes were an important determinant of SARS-CoV-2 cell entry of host and severity of the disease. In this point of view, reversible nature of epigenetic modifications holds promise for future studies that may block the infection activity of the virus. Our study sheds light on future studies that should focus on the role of these epigenetic modifications in infection outcome, the severity of the disease, and entrance of host cell.
